# Impact of adrenaline and metabolic stress on exercise‐induced intracellular signaling and PGC‐1*α* mRNA response in human skeletal muscle

**DOI:** 10.14814/phy2.12844

**Published:** 2016-07-19

**Authors:** Nina Brandt, Thomas P. Gunnarsson, Morten Hostrup, Jonas Tybirk, Lars Nybo, Henriette Pilegaard, Jens Bangsbo

**Affiliations:** ^1^ The August Krogh CentreSection for Cell Biology and PhysiologyDepartment of BiologyUniversity of CopenhagenCopenhagenDenmark; ^2^ Section for Integrated PhysiologyDepartment of NutritionExercise and SportsUniversity of CopenhagenCopenhagenDenmark; ^3^ Bispebjerg University HospitalCopenhagenDenmark

**Keywords:** Exercise, human muscle biopsies, intracellular signaling, metabolic stress, PGC‐1*α *mRNA

## Abstract

This study tested the hypothesis that elevated plasma adrenaline or metabolic stress enhances exercise‐induced PGC‐1*α* mRNA and intracellular signaling in human muscle. Trained (VO_2_‐max: 53.8 ± 1.8 mL min^−1^ kg^−1^) male subjects completed four different exercise protocols (work load of the legs was matched): C – cycling at 171 ± 6 W for 60 min (control); A – cycling at 171 ± 6 W for 60 min, with addition of intermittent arm exercise (98 ± 4 W). DS – cycling at 171 ± 6 W interspersed by 30 sec sprints (513 ± 19 W) every 10 min (distributed sprints); and CS – cycling at 171 ± 6 W for 40 min followed by 20 min of six 30 sec sprints (clustered sprints). Sprints were followed by 3:24 min:sec at 111 ± 4 W. A biopsy was obtained from m. vastus lateralis at rest and immediately, and 2 and 5 h after exercise. Muscle PGC‐1*α* mRNA content was elevated (*P* < 0.05) three‐ to sixfold 2 h after exercise relative to rest in C, A, and DS, with no differences between protocols. AMPK and p38 phosphorylation was higher (*P* < 0.05) immediately after exercise than at rest in all protocols, and 1.3‐ to 2‐fold higher (*P* < 0.05) in CS than in the other protocols. CREB phosphorylation was higher (*P* < 0.05) 2 and 5 h after exercise than at rest in all protocols, and higher (*P* < 0.05) in DS than CS 2 h after exercise. This suggests that neither plasma adrenaline nor muscle metabolic stress determines the magnitude of PGC‐1*α* mRNA response in human muscle. Furthermore, higher exercise‐induced changes in AMPK, p38, and CREB phosphorylation are not associated with differences in the PGC‐1*α* mRNA response.

## Introduction

Endurance exercise increases the oxidative capacity of skeletal muscle through increased mitochondrial biogenesis and vascularization (Booth et al. 2012). This is exemplified by increases in the content of proteins in the respiratory chain (Hood 1985) cytochrome (cyt) c, and the regulator of angiogenesis, vascular endothelial growth factor (VEGF), in human skeletal muscle with exercise (Gustafsson et al. 1985; Baar et al. 2002; Olesen et al. 2014, 2015). Mechanisms underlying such long‐term adaptations have been suggested to include cumulative effects of transient gene responses to each single exercise bout (Neufer et al. 1996). In accordance, studies have reported exercise‐induced transient increases in both Cyt c and VEGF mRNA in human skeletal muscle (Hiscock et al. 2003; Jensen et al. 2004; Leick et al. 2010).

The transcriptional coactivator peroxisome proliferator‐activated receptor‐ϒ coactivator (PGC‐1*α*), which is known as a key regulator of mitochondrial biogenesis and vascularization, has been suggested to elicit exercise‐induced transcriptional regulation of oxidative and angiogenic factors in skeletal muscle. In addition, PGC‐1*α* transcription and mRNA content in skeletal muscle have been shown to be upregulated after a single exercise bout in rats (Baar et al. 2002), mice (Leick et al. 2008), and humans (Pilegaard et al. 2003). Therefore, it has been suggested that exercise‐induced transcriptional regulation of PGC‐1*α* is an initial event in the concomitant regulation of oxidative and angiogenic markers in skeletal muscle.

The PGC‐1*α* mRNA has been shown to increase in human skeletal muscle after both prolonged moderate intensity exercise and brief intense interval exercise (Gibala et al. 1985), and the exercise‐induced PGC‐1*α* mRNA response in human skeletal muscle has been demonstrated to be intensity dependent (Egan et al. 2010; Nordsborg et al. 2010). However, it is unclear how exercise‐induced PGC‐1*α* transcription is regulated. PGC‐1*α* transcription is believed to be regulated via the CaMK/cAMP‐response element binding (CREB) pathway (Handschin et al. 2003) or p38 mitogen‐activated protein kinase (p38) (Akimoto et al. 2005; Wright et al. 2007). In accordance, p38 and CREB have been reported to be phosphorylated and thereby activated in skeletal muscle by contractile activity in mice (Akimoto et al. 2005; Bruno et al. 2014) and humans (Boppart et al. 2000; Egan et al. 2010). Furthermore, the cAMP‐response element and the binding sites of the suggested p38 targets MEF2 and ATF2 have been located in the PGC‐1*α* promoter, further supporting that CREB and p38 contribute in the regulation of PGC‐1*α* transcription. An autoregulatory loop controls PGC‐1*α* expression in skeletal muscle (Handschin et al. 2003), and exercise stimulates PGC‐1*α* transcription through activation of the p38 MAPK pathway (p38) (Akimoto et al. 2005). Moreover, p38 has been reported to phosphorylate PGC‐1*α* at three residues leading to a more active PGC‐1*α* protein (Puigserver et al. 2001), which may indicate that p38 regulates PGC‐1*α* activity in response to exercise. In accordance, a human study has reported that PGC‐1*α* mRNA induction was preceded by p38 signaling in skeletal muscle (Gibala et al. 1985). However, whether differences in exercise‐induced PGC‐1*α* mRNA responses are associated with differences in the potential upstream PGC‐1*α* regulators, p38 and CREB, remain unresolved.

The finding that adrenaline injections increased PGC‐1*α* mRNA in mouse skeletal muscle (Chinsomboon et al. 2009) suggests that adrenaline may play a role in exercise‐induced PGC‐1*α* transcriptional regulation. This is further supported by studies showing that injections of the beta2‐adrenoceptor agonist clenbuterol increased PGC‐1*α* mRNA more than 30‐fold in mouse skeletal muscle (Miura et al. 2007; Chinsomboon et al. 2009) and pretreatment with the beta‐antagonist propranolol inhibited this increase (Miura et al. 2007). Similarly, an exercise‐induced increase in PGC‐1*α* mRNA in mouse skeletal muscle was almost blunted by pretreatment with propranolol (Miura et al. 2007). However, a role of adrenaline in exercise‐induced regulation of PGC‐1*α* expression in humans is less clear. Beta‐antagonist blockade during endurance exercise has been reported to blunt the increase in maximal activity of selected oxidative enzymes (Svedenhag et al. 1984; Wolfel et al. 1986), while another human study did not observe alterations in markers of mitochondrial biogenesis with beta2‐adrenergic stimulation (Robinson et al. 2010).

The observation that PGC‐1*α* mRNA remains elevated in human skeletal muscle 8 h after exercise when muscle glycogen content is maintained low through diet manipulation (Pilegaard et al. 2005) may suggest that the intracellular energy store also affects the PGC‐1*α* mRNA level. This is further supported by the augmented PGC‐1*α* mRNA response when exercise is performed with reduced muscle glycogen (Psilander et al. 2013). Together, these findings may indicate that the intracellular energy state also influences PGC‐1*α* transcription in skeletal muscle. AMP‐activated protein kinase (AMPK) is known as an intracellular energy sensor, activated by phosphorylation during exercise (Winder and Hardie 1996). In addition, the observation that an exercise‐induced PGC‐1*α* mRNA induction was preceded by increased AMPK phosphorylation in human skeletal muscle (Gibala et al. 1985), and the identification of a DNA sequence mediating transcriptional regulation of PGC‐1*α* during AMPK activation (Irrcher et al. 2008) further support this possibility. Finally, in vitro experiments have indicated that AMPK phosphorylates PGC‐1*α* on two residues (Jager et al. 2007) suggesting that AMPK also regulates PGC‐1*α* activity in skeletal muscle in response to exercise.

The present study tested the hypothesis that elevated levels of plasma adrenaline or metabolic stress enhances PGC‐1*α* mRNA responses in human skeletal muscle with concomitant effects on downstream targets, and that difference in AMPK, p38, and CREB signaling between the different exercise modalities are associated with corresponding differences in PGC‐1*α* mRNA responses.

## Materials and methods

### Subjects

The study comprised 10 moderately trained male subjects with an average age of 25.8 ± 5.9 years (mean ± SD) (range: 21–34 years), body mass 81.3 ± 9.4 (57.7–91.1) kg, height 184 ± 6 (170–190) cm, body mass index 23.9 ± 2.0 (20.0–26.8) kg m^−2^, and maximum oxygen uptake (VO_2_‐max) 53.8 ± 5.7 (46.2–60.6) mL kg^−1^ min^−1^. The subjects were engaged in 1–3 weekly training sessions (team sports, endurance, and/or strength training). Subjects were informed of any risks and discomforts associated with the experiments before giving their written informed consent to participate. The study was approved by the Ethics Committee of Copenhagen and Frederiksberg communities and adheres to the principles of the Declaration of Helsinki and Title 45, U.S. Code of Federal Regulations, Part 46, Protection of Human Subjects, Revised 23 June 2005, effective 23 June 2005.

### Study design

The study was designed as a randomized crossover study comparing four different exercise protocols conducted at separate experimental days. The study period was 24 ± 2 days (19–41 days). At least 48 h prior to the first experimental day, subjects were interviewed and completed a VO_2_‐max test consisting of a 5‐min cycling bout at 100–150 W followed by 5 min at 175–225 W (depending on fitness level), and then, work load was increased by 25 W min^−1^ until volitional exhaustion. If pedaling frequency dropped below 50 rpm, the test was terminated immediately. The incremental peak power output (iPPO) was calculated based on the performance during the incremental test. Pulmonary oxygen uptake was measured (Oxycon Pro, Viasys Healthcare, Hoechberg, Germany) throughout the test and VO_2_‐max was determined as the highest value achieved during a 30‐s period. Criteria used for achievement of VO_2_‐max were a plateau in VO_2_ despite an increased workload and a respiratory exchange ratio above 1.10. VO_2_ during sub‐ and maximal cycling was used to calculate individual workloads (60% of VO_2_‐max) on experimental days.

#### Experimental period

**Figure 1:**
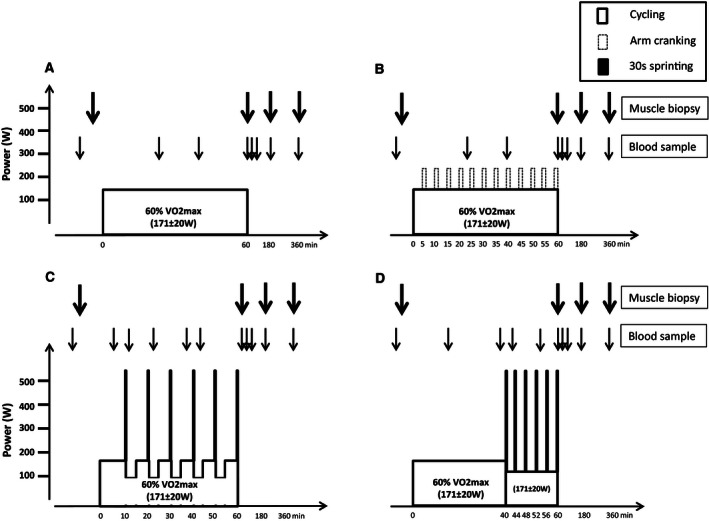
Schematic presentation of the four experimental days consisting of 60 min of cycling at an average intensity corresponding to 60% of VO_2_‐max (171 ± 6 W) as (A) continuous cycling (C), (B) continuous cycling with intermittent arm exercise (A), (C) continuous cycling interspersed by six 30 sec sprints (513 ± 19 W) and cycling at 111 ± 4 W for 3:24 min:sec every 10 min (DS), and (D) 40 min of cycling followed by 20 min with six 30 sec sprints (513 ± 19 W) interspersed by 3:24 min:sec at 111 ± 4 W (CS). On experimental days, muscle biopsies were taken at rest as well as immediately, and 120 and 300 min after exercise. Blood samples were taken at rest, during (23, 39, and 60 min) and after (3, 10, 120, and 300 min) exercise.

First experimental day was commenced 6 ± 1 days after the screening. The order of experimental days was randomized. During the experimental period subjects maintained their habitual lifestyle and training routines. The four experimental days consisted of either continuous cycling at an intensity corresponding to 60% VO_2_‐max (171 ± 6 W) with or without addition of intermittent arm cranking, interspersed or followed by clustered repeated 30 sec sprints (513 ± 19 W) (Fig. 1):

C: 60 min of continuous cycling at 171 ± 6 W (control).

A: 60 min of continuous cycling at 171 ± 6 W with addition of twelve 1 min bouts (98 ± 4 W) of intermittent arm cranking every 5 min (additional arm exercise).

DS: 60 min of continuous cycling at 171 ± 6 W interspersed by 30 sec sprints (513 ± 19 W) and cycling at 111 ± 4 W for 3:24 min:sec every 10 min (distributed sprints).

CS: 40 min of continuous cycling at 171 ± 6 W followed by 20 min consisting of six 30 sec sprints (513 ± 19 W) interspersed by 3:24 min:sec at 111 ± 4 W (clustered sprints).

The workload of the legs was matched between experimental days, eliciting an average intensity of 60% of VO_2_‐max (171 ± 6 W). In A, the average heart rate (HR) and pulmonary oxygen uptake (VO_2_) were higher than in the other protocols, due to addition of intermittent arm cranking (98 ± 4 W) (Table 2).

The rationale for choosing the specific exercise protocols were that C was control cycling, with a very modest anaerobic energy contribution, thus limited stimulation of adrenaline production, utilization of muscle creatine phosphate (CP), accumulation of muscle lactate, and reduced muscle pH. In A, we included 1 min arm‐exercise bouts every 5 min (twelve in total) to stimulate the production of adrenaline. With the distributed sprint protocol (DS) we wanted to induce temporary reductions in muscle CP, accumulation of muscle lactate and decreases in muscle pH. The clustered sprint protocol (CS) was to induce a marked metabolic response, with repeated bouts of high utilization of muscle CP, accumulation of muscle lactate, and reduction in muscle pH.

On experimental days, subjects reported to the laboratory in the morning (between 8 and 9 am) 2 h after consumption of a self‐chosen standardized breakfast. During the 60 min of exercise, pulmonary VO_2_ was measured (Oxycon Pro, Viasys Healthcare, Hoechberg, Germany) at 5–10 and 39–45 min in C, at 3–11 and 38–46 min in A, at 4–14 and 37–44 min in DS, and at 5–10 and 37–46 min in CS. In addition, HR was measured (Polar team system, Polar, Electro Oy) throughout the exercise protocol.

A muscle biopsy was collected at rest, immediately, and 2 and 5 h after exercise using the percutaneous needle biopsy technique (Bergstrom 1975) with suction. Within 60 min of arrival at the laboratory, local anesthesia (1 mL of lidocaine, 20 mg mL^−1^ without adrenaline) was administered over m. vastus lateralis, and two 3‐mm incisions were made. A resting muscle biopsy (rest) was obtained from the most distal incision, and the second incision was used for a second muscle biopsy sampled immediately after exercise (0 h). Additional muscle biopsies were sampled 2 and 5 h into recovery in the opposite leg through separate incisions. The muscle biopsy samples were immediately frozen in liquid nitrogen and stored (−80°C) until analysis. Blood samples were taken from a catheter (18 gauge, 32 mm) inserted in an antecubital vein prior to exercise. A blood sample was collected at rest, at 23, 39, and 60 min of exercise and 3, 10, 120, and 300 min after exercise.

### Analyses

#### Blood and muscle analyses

For the determination of plasma lactate and glucose, blood samples were drawn in heparinized 2 mL syringes and immediately analyzed (ABL 800 Flex, Radiometer, Copenhagen). For the analyses of plasma catecholamines (Plasma ELISA High Sensitive kit, LDN, Nordhorn, Germany), blood samples were collected in 5 mL syringes and transferred to an Eppendorf tube containing 30 *μ*L EDTA (0.2 mol/L), after which they were spun at 20,000*g* for 2 min to collect plasma, which was stored at −20°C until analysis.

Approximately 20 mg of the muscle samples were frozen separately for mRNA analyses. The remaining part of the muscle sample was immediately frozen in liquid nitrogen and stored at −80°C before being freeze dried for 48 h and dissected free of all nonmuscle elements. Dissection was performed under a stereo microscope with an ambient temperature of ~18°C and a relative humidity below 30%. After dissection, muscle tissue was weighed and separated into different tubes for analyses.

#### Muscle glycogen

Muscle glycogen was determined on freeze‐dried muscle tissue (~2 mg dw) before (rest) as well as immediately, and 2 and 5 h after exercise by acid hydrolysis at 100°C for 2 h followed by determination of glycosyl units using the hexokinase method (Lowry and Passonneau 1972).

#### Muscle Metabolites

Muscle lactate, ATP, and CP were determined on freeze‐dried muscle tissue (~2 mg dry wt) before (rest) as well as immediately, and 2 and 5 h after exercise by extraction in 3 mol/L perchloric acid followed by fluorometric analyses as described previously (Lowry and Passonneau 1972).

#### Muscle proteins

Muscle lysate was produced from ~4 to 6 mg dry weight by homogenization in an ice‐cold buffer (10% glycerol, 20 mmol/L Na‐pyrophosphate, 150 nmol/L NaCl, 50 mmol/L HEPES, 1% NP‐40, 20 mmol/L *β*‐glycerophosphate, 10 mmol/L NaF, 1 mmol/L EDTA, 1 mmol/L EGTA, 20 *μ*g/mL aprotinin, 10 *μ*g/mL leupeptin, 2 mmol/L Na_3_VO_4_, 3 mmol/L benzamidine, pH 7.5) for 2 min at 30 oscillations per second in a TissueLyser (TissueLyser II, Qiagen, Valencia, CA, USA). The samples were set to rotate end over end for 1 h at 4°C followed by centrifugation at 17,500*g* for 20 min at 4°C. The lysates were collected as the supernatant. The protein content in the lysates was determined by the bicinchoninic acid method (Pierce Chem, Comp., IL) and lysates were prepared with sample buffer containing sodium dodecyl sulfate (SDS) and boiled for 3 min at 96°C. Phosphorylation levels and protein content were measured by SDS‐PAGE and western blotting using self‐casted gels. Specific amounts of total protein were loaded for each sample. PVDF membranes were blocked in 3% fish gel. Primary antibodies used were phospho‐AMPK (#2535S, Cell Signaling), AMPK*α*2 protein (#G3013, Santa Cruz Biotechnology), phospho‐ACC (07‐303, Millipore), phospho‐p38 (#4511, Cell Signaling), P38 protein (#9212, Cell Signaling), phospho‐CREB (#9191, Cell Signaling), and CREB protein (#9197, Cell Signaling). Secondary antibodies were HRP conjugated (Dako, Glostrup, Denmark). Luminata^™^ Classico Western HRP Substrate (Millipore, Denmark) was used to detect protein and phosphorylation. Band intensity was quantified using ImageQuant Las 4000 (GE Healthcare, Munich, Germany) and ImageQuant Imaging software. Protein content and phosphorylation were expressed in arbitrary units relative to control samples loaded on each site of each gel. Phosphorylation levels were normalized to total protein content of the target protein.

#### RNA isolation, reverse transcription, and real‐time PCR

Total RNA was isolated from 15 to 20 mg muscle tissue (wet weight) by a modified guanidinium thiocyanate–phenol–chloroform extraction method from Chomczynski and Sacchi (1987) as described previously (Pilegaard et al. 2000) except for the use of a TissueLyser (TissueLyser II, Qiagen, Valencia, CA, USA) for homogenization.

Superscript II RNase H‐system and Oligo dT (Invitrogen, Carlsbad, CA, USA) were used to reverse transcribe the mRNA to cDNA as described previously (Pilegaard et al. 2000).

Quantification of cDNA as a measure of mRNA content of a given gene was performed by real‐time PCR using an ABI 7900 sequence‐detection system (Applied Biosystems, Foster City, CA, USA). Primers and TaqMan probes were designed from human specific databases from ensemble (www.ensembl.org/Homo_sapiens/Info/Index) and Primer Express (Applied Biosystems) and are presented in Table 1. Self‐designed TaqMan probes were labeled with 5′‐6‐carboxyfluorescein (FAM) and 3′‐6‐carboxy‐N,N,N′,N′‐tetramethylrhodamine (TAMRA) and obtained from TAG Copenhagen (Copenhagen, Denmark). Cyclophilin A mRNA was amplified using a 5′‐VIC‐ and 3′‐TAMRA‐labeled predeveloped assay reagent (Applied Biosystems). Cyclophilin A was used as endogenous control as there was no significant difference between protocols and/or time points. Real‐time PCR was performed in triplicates in a total reaction volume of 10 *μ*L using Universal Mastermix with UNG (Applied Biosystems). The obtained cycle threshold values reflecting the initial content of the specific transcript in the samples were converted to a relative amount by using standard curves constructed from serial dilution of a pooled sample made from all samples. For each cDNA sample, the mRNA content of the given target was normalized to Cyclophilin A mRNA.

**Table 1:** Primers and TaqMan probe sequences used for real‐time PCR

	Forward primer	Reverse primer
PGC‐1*α*	5′‐CAAGCCAAACCAACAACTTTATCTCT‐3′	5′‐CACACTTAAGGTGCGTTCAATAGTC‐3′
VEGF	5′‐CTTGCTGCTCTACCTCCACCAT‐3′	5′‐ATGATTCTGCCCTCCTCCTTCT‐3′
Cyt c	5′‐GGTCTCTTTGGGCGGAAGAC‐3′	5′‐CTCTCCCCAGATGATGCCTTT‐3′
TaqMan probe		
PGC‐1*α*	5′‐AGTCACCAAATGACCCCAAGGGTTCC‐3′	
VEGF	5′‐AAGTGGTCCCAGGCTGCACCCA‐3′	
Cyt c	5′‐CCCTGGATACTCTTACACAGCCGCCAA‐3′	

PGC‐1*α*, peroxisome proliferator‐activated receptor‐ϒ coactivator‐1*α*; VEGF, vascular endothelial growth factor; Cyt c, cytochrome c.

### Statistics

Values are presented as mean (±SE). All subjects (*n* = 10) contributed to blood analyses, muscle glycogen, ATP, creatine phosphate, lactate, and protein analysis, while eight subjects contributed to the mRNA analyses due to extremely low RNA yield in two of the subjects. Two‐way analysis of variance for repeated measures was applied to evaluate the effect of protocol and time points on plasma adrenaline, muscle glycogen, muscle ATP, muscle creatine phosphate, muscle lactate, mRNA, phosphorylation levels, and protein content. If a main effect was observed, a Student–Newman–Keul's post hoc test was used to locate differences. A significant level of *P* < 0.05 was chosen, and statistical calculations were performed using SigmaPlot Version 12.5. Normality was tested before each statistical test. Sample power analysis was performed prior to the study with PGC‐1*α* mRNA, a fold change of 2, a power of 0.8, and *α *= 0.05 giving a minimum required sample size of *n* = 8. Based on previous experiments, we expected a dropout rate of up to 20%, thus recruiting 10 subjects for this experiment.

## Results

### Heart rate and oxygen uptake

Heart rate (HR) after 23 min of exercise was lower (*P* < 0.05) in C, DS, and CS than A, and lower after 39 and 60 min (*P* < 0.05) in C and CS than A (Table 2). In addition, HR after 60 min was lower (*P* < 0.05) in C and CS than DS and lower (*P* < 0.05) in C than CS. There was no difference in pulmonary oxygen uptake between protocols after 9 and 39 min of exercise (Table 2).

**Table 2:** Heart rate (HR) and pulmonary oxygen uptake ($\dot{V}O_{2}$) during 60 min of cycling exercise (*n* = 10) as continuous cycling (C), continuous cycling with arm exercise (A), continuous cycling with sprints performed every 10 min (DS), and continuous cycling followed by repeated sprints for 20 min (CS)

	Exercise (min)	C	A	DS	CS
HR (bpm)	23	143 ± 5^a^	157 ± 5	140 ± 6^a^	143 ± 3^a^
	39	149 ± 6^a^	161 ± 5	155 ± 5	149 ± 3^a^
	60	155 ± 5^c^	177 ± 5	172 ± 4	164 ± 4^a^^,^ b
$\dot{V}O_{2}$ (L/min)	9	2.68 ± 0.07^a^	2.85 ± 0.10	2.72 ± 0.07^a^	2.75 ± 0.08^a^
	39	2.86 ± 0.07^a^	2.94 ± 0.11	2.76 ± 0.08^a^	2.78 ± 0.08^a^

Values are presented as mean ± SE

a Different (*P* < 0.05) from A.

b Different (*P* < 0.05) from DS

c Different (*P* < 0.05) from all other protocols.

### Plasma adrenaline

**Figure 2:**
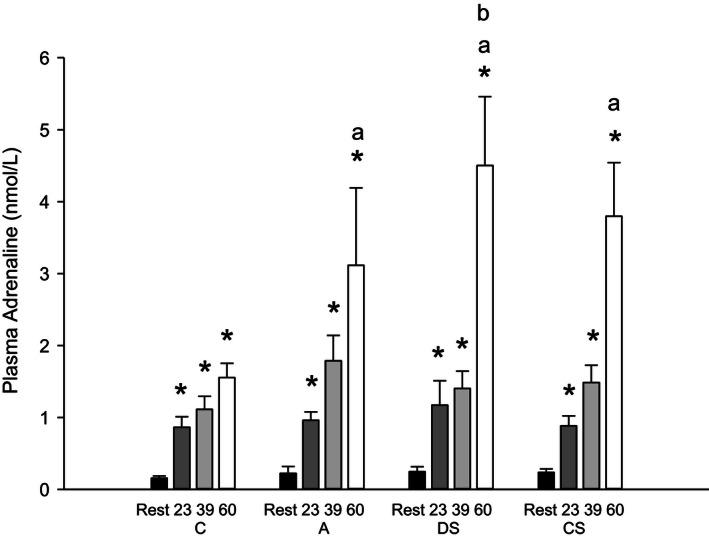
Venous plasma adrenaline concentrations before (Rest; black bars), during (23 min: dark gray bars; 39 min: light gray bars; 60 min; white bars) 60 min of cycling at 171 ± 6 W on average as continuous control cycling (C), continuous cycling with arm exercise (A), continuous cycling interspersed by six 30 sec sprints (513 ± 19 W) and cycling at 111 ± 4 W for 3:24 min:sec every 10 min (DS), and 40 min of control cycling followed by 20 min with six 30 sec sprints (513 ± 19 W) interspersed by 3:24 min:sec at 111 ± 4 W (CS). Values (*n* = 10) are presented as mean ± SE. *Different (*P* < 0.05) from rest. ^a^Different (*P* < 0.05) from C. ^b^Different (*P* < 0.05) from A.

Plasma adrenaline after 23 and 39 min of exercise was not different between protocols, whereas plasma adrenaline was higher (*P* < 0.05) after 60 min of exercise in A, DS, and CS than in C, and higher (*P* < 0.05) in DS than in A (Fig. 2).

### Plasma lactate and glucose

Plasma lactate after 23 and 39 min of exercise was higher (*P* < 0.05) in DS and A than in C, and higher (*P* < 0.05) in DS than in A (Table 3). At the end of exercise, plasma lactate was higher (*P* < 0.05) in CS than the other protocols, and higher (*P* < 0.05) in A and DS than in C. Plasma lactate remained higher in A, DS, and CS after 10 but not 120 min of recovery.

**Table 3:** Plasma glucose (mmol/L) and lactate (mmol/L) before (Rest), during and after 60 min of exercise (*n* = 10) as continuous cycling (C), continuous cycling with arm exercise (A), continuous cycling with sprints performed every 10 min (DS), and continuous cycling for 40 min followed by repeated sprints for 20 min (CS)

	Glucose (mmol/L)				Lactate (mmol/L)			
	C	A	DS	CS	C	A	DS	CS
Rest	5.8 ± 0.3	5.6 ± 0.3	5.4 ± 0.2	5.5 ± 0.2	1.1 ± 0.2	1.1 ± 0.2	1.5 ± 0.3	1.1 ± 0.1
Exercise (min)	C	A	DS	CS	C	A	DS	CS
23	4.6 ± 0.2^a^	5.0 ± 0.2	5.6 ± 0.2^e^	5.0 ± 0.1	2.4 ± 0.9	5.9 ± 0.8^a^, ^b^^,^ d	9.4 ± 1.9^a^^,^ e	2.5 ± 0.6
39	5.1 ± 0.1	5.5 ± 0.2	5.8 ± 0.4	5.3 ± 0.1	2.4 ± 0.8	6.1 ± 0.9^a^, ^b^^,^ d	6.0 ± 2.0^a^, ^b^^,^ d	2.1 ± 0.4
60	5.2 ± 0.1	5.9 ± 0.2^b^	5.7 ± 0.3	6.7 ± 0.3^a^, ^e^	2.3 ± 0.6	7.3 ± 0.8^a^, ^b^	8.9 ± 1.6^a^, ^b^	13.2 ± 1.5^a^, ^e^
Recovery (min)	C	A	DS	CS	C	A	DS	CS
3	5.6 ± 0.1	6.3 ± 0.2^b^	6.3 ± 0.4^a^, ^b^	7.0 ± 0.4^a^, ^e^	1.8 ± 0.3	6.7 ± 0.9^a^, ^b^	10.7 ± 1.7^a^, ^b^^,^ c	13.7 ± 1.5^a^, ^e^
10	5.5 ± 0.2	6.0 ± 0.2	5.7 ± 0.5	6.6 ± 0.3^a^, ^e^	1.6 ± 0.5	4.9 ± 0.8^a^, ^b^	7.5 ± 1.5^a^, ^b^^,^ c	11.3 ± 1.6^a^, ^e^
120	5.3 ± 0.1	5.2 ± 0.1	5.1 ± 0.1	5.2 ± 0.1	0.8 ± 0.1	0.9 ± 0.1	1.2 ± 0.2	1.3 ± 0.1
300	5.1 ± 0.1	5.1 ± 0.1	5.0 ± 0.2	5.1 ± 0.1	0.7 ± 0.1	0.8 ± 0.1	0.9 ± 0.1	0.9 ± 0.1

Values are presented as mean ± SE.

a Different (*P* < 0.05) from rest.

b Different (*P* < 0.05) from C.

c Different (*P* < 0.05) from A.

d Different (*P* < 0.05) from CS.

e Different (*P* < 0.05) from all other protocols.

In C, plasma glucose after 23 min of exercise was lower (*P* < 0.05) than at rest. After 39 min of exercise, plasma glucose was not different between protocols, whereas plasma glucose was higher (*P* < 0.05) in CS immediately, 3 and 10 min after exercise than in the other protocols, and 3 min after exercise it was higher (*P* < 0.05) in A and DS than C.

### Muscle ATP and CP

Muscle ATP before and after exercise was not different between protocols. Muscle CP at the end of exercise was lower (*P* < 0.05) in DS and CS than in C and A (Table 4).

**Table 4:** Muscle ATP, creatine phosphate (CP), glucose, glucose‐6‐phosphate (G‐6‐P) and lactate (mmol∙kg dw^−1^) before (Pre) and immediately after (Post) 60 min of continuous cycling exercise (*n* = 10) consisting of 60 min of continuous cycling (171 ± 20 W) (C), 60 min of exercise (*n* = 10) as continuous cycling (C), continuous cycling with arm exercise (A), continuous cycling with sprints performed every 10 min (DS), and continuous cycling for 40 min followed by repeated sprints for 20 min (CS)

	C		A		DS		CS	
	Pre	Post	Pre	Post	Pre	Post	Pre	Post
ATP	25.3 ± 1.5	23.3 ± 1.0	23.9 ± 1.3	23.1 ± 1.5	26.7 ± 0.8	24.2 ± 1.5	25.9 ± 1.7	23.4 ± 1.4
CP	73.3 ± 3.1	49.5 ± 4.1^a^	73.5 ± 3.3	46.9 ± 6.4^a^	78.9 ± 20.7	20.7 ± 4.1^a^, ^b^^,^ c	74.1 ± 5.0	26.7 ± 4.3^a^, ^b^^,^ c
Glucose	2.3 ± 0.4	2.5 ± 0.4	2.8 ± 0.2	2.8 ± 0.5	1.7 ± 0.3	6.9 ± 1.4^a^, ^b^^,^ c	1.4 ± 0.1	14.0 ± 2.1^a^, ^d^
G‐6‐P	1.3 ± 0.6	1.8 ± 0.6	2.3 ± 0.9	3.0 ± 0.7	1.1 ± 0.4	4.0 ± 1.3^a^	1.0 ± 0.2	5.7 ± 1.5^a^, ^b^^,^ c
Lactate	4.4 ± 1.6	12.2 ± 3.0^a^	9.6 ± 3.3	17.1 ± 3.2^a^	4.2 ± 1.5	62.4 ± 5.2^a^, ^b^^,^ c	3.7 ± 1.0	74.3 ± 5.2^a^, ^d^

Values are presented as mean ± SE.

a Different (*P* < 0.01) from rest.

b Different (*P* < 0.05) from C.

c Different (*P* < 0.05) from A.

d Different (*P* < 0.05) from all other protocols.

### Muscle glycogen, glucose, glucose‐6‐phosphate, and lactate

**Figure 3:**
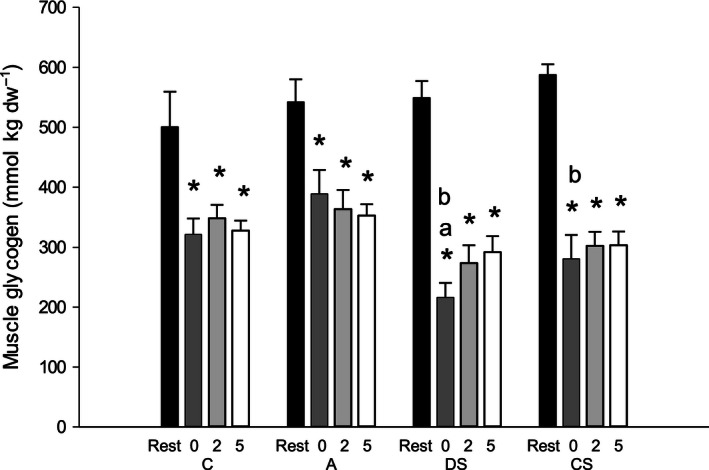
Muscle glycogen levels (mmol∙kg dw^−1^) before (rest; black bars), immediately (0 h; dark gray bars), 2 h (light gray bars), and 5 h (white bars) after 60 min of cycling (171 ± 6 W) as continuous control cycling (C), continuous cycling with arm exercise (A), continuous cycling interspersed by six 30 sec sprints (513 ± 19 W) and cycling at 111 ± 4 W for 3:24 min:sec every 10 min (DS), and 40 min of control cycling followed by 20 min with six 30 sec sprints (513 ± 19 W) interspersed by 3:24 min:sec at 111 ± 4 W (CS). Values (*n* = 10) are presented as mean±SE. *Different (*P* < 0.05) from rest. ^a^Different (*P* < 0.05) from C. ^b^Different (*P* < 0.05) from A.

Muscle glycogen in DS was lower (*P* < 0.05) than C and A, and lower (*P* < 0.05) in CS than A at the end of exercise (Fig. 3). Muscle glycogen 2 and 5 h after exercise was not different between protocols.

In CS, muscle glucose after exercise was higher (*P* < 0.05) than in the other protocols, and higher (*P* < 0.05) in DS than in C and A. In DS, muscle glucose‐6‐phosphate (G‐6‐P) was higher (*P* < 0.05) than in C and A at the end of exercise (Table 4). At the end of exercise, muscle lactate was higher (*P* < 0.05) in CS than in the other protocols, and higher (*P* < 0.05) in DS than in C and A (Table 4).

### Muscle mRNA levels

**Figure 4:**
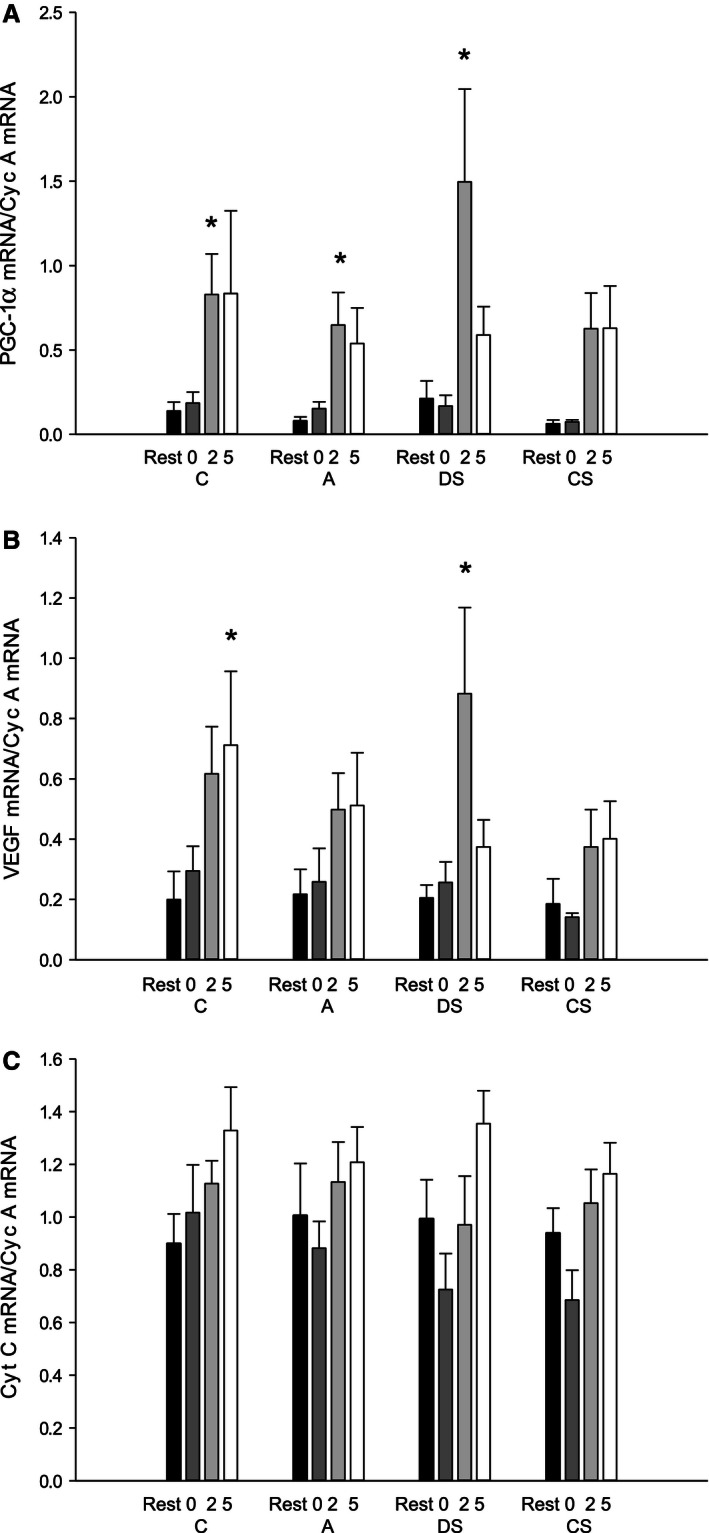
(A) Peroxisome proliferator‐activated receptor‐ϒ coactivator‐1*α* (PGC‐1*α*), (B) vascular endothelial growth factor (VEGF), and (C) cytochrome c (Cyt C) mRNA content normalized to cyclophilin A mRNA content in vastus lateralis before (rest; black bars), immediately (0 h; dark gray bars), 2 h (light gray bars), and 5 h (white bars) after 60 min of cycling (171 ± 6 W) as continuous control cycling (C), continuous cycling with arm exercise (A), continuous cycling interspersed by six 30 sec sprints (513 ± 19 W) and cycling at 111 ± 4 W for 3:24 min:sec every 10 min (DS), and 40 min of control cycling followed by 20 min with six 30 sec sprints (513 ± 19 W) interspersed by 3:24 min:sec at 111 ± 4 W (CS). Values are presented as mean±SE;* n* = 8. *Different (*P* < 0.05) from rest.

Muscle PGC‐1*α* mRNA content was elevated (*P* < 0.05) three‐ to sixfold relative to rest 2 h after exercise in C, A, and DS with no differences between protocols (Fig. 4A).

Muscle VEGF mRNA content was 1‐ to 3.2‐fold higher (*P* < 0.05) relative to rest 2 h after exercise in DS (*P* < 0.05) and 5 h after exercise in C (*P* < 0.05), with no differences between protocols (Fig. 4B). Cyt c mRNA was unaffected by the exercise bout in all protocols with no differences between protocols (Fig. 4C).

### Protein content and phosphorylation status of skeletal muscle

**Figure 5:**
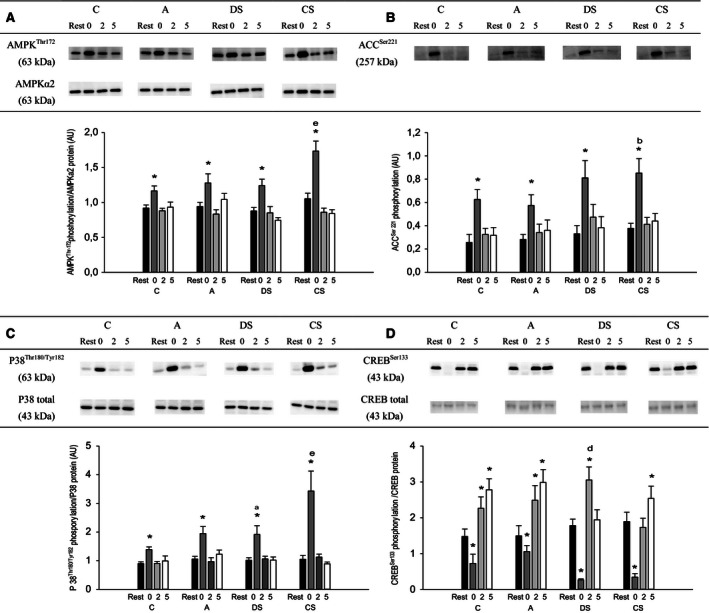
(A) AMP‐activated protein kinase (AMPK)^Thr172^ phosphorylation normalized to AMPK *α*2 protein, (B) acetyl‐CoA carboxylase (ACC)^Ser79^ phosphorylation, (C) p38 mitogen‐activated protein kinase (p38)^Thr180/Tyr182^ phosphorylation normalized to p38 protein content, and (D) cAMP‐response element‐binding protein (CREB)^Ser133^ phosphorylation normalized to total CREB protein content before (rest; black bars), immediately (0 h; dark gray bars), 2 h (light gray bars), and 5 h (white bars) after 60 min of cycling (171 ± 6 W) as continuous control cycling (C), continuous cycling with arm exercise (A), continuous cycling interspersed by six 30 sec sprints (513 ± 19 W) and cycling at 111 ± 4 W for 3:24 min:sec every 10 min (DS), and 40 min of control cycling followed by 20 min with six 30 s sprints (513 ± 19 W) interspersed by 3:24 min:s at 111 ± 4 W (CS). Protein levels are given in arbitrary units (AU). Values are presented as mean±SE;* n* = 10. *Different (*P* < 0.05) from rest. ^a^Different (*P* < 0.05) from C. ^b^Different (*P* < 0.05) from A. ^d^Different (*P* < 0.05) from CS. ^e^Different (*P* < 0.05) from all other protocols.

In CS, AMPK*α*2 protein content was higher (*P* < 0.05) 0, 2, and 5 h after exercise than at rest with no differences in the other protocols. No difference in AMPK*α*2 protein was observed between protocols (Fig. 5A). In all protocols, AMPK and ACC phosphorylation was higher (*P* < 0.05) at the end of exercise than at rest, and AMPK phosphorylation was ~1.3‐fold higher (*P* < 0.05) in CS than in the other protocols (Fig. 5A and 5B). In addition, ACC phosphorylation immediately after exercise was 1.3‐fold higher (*P* < 0.05) in CS than in A, but not different from C and DS.

There were no changes in p38 protein content in any of the protocols, but p38 protein content was overall lower (*P* < 0.05) in CS than in the other protocols. At the end of exercise p38 phosphorylation was higher (*P* < 0.05) than at rest in all protocols, and p38 phosphorylation was 1.3‐fold higher (*P* < 0.05) in DS than in C and 1.6‐ to 2‐fold higher (*P* < 0.05) in CS than the other protocols (Fig. 5C).

There was no effect of exercise or difference between protocols in CREB content. In all protocols, CREB phosphorylation was lower (*P* < 0.05) immediately after exercise than at rest with no difference between protocols (Fig. 5D). CREB phosphorylation was higher (*P* < 0.05) 2 h after exercise than at rest in C, A, and DS and higher (*P* < 0.05) 5 h after exercise than at rest in C, A, and CS. CREB phosphorylation was higher (*P* < 0.05) in DS than in CS 2 h after exercise.

## Discussion

The main findings of the present study were that neither differences in plasma adrenaline levels nor differences in muscle lactate, glycogen, and creatine phosphate affected the exercise‐induced PGC‐1*α* mRNA response in human skeletal muscle. In addition, differences in exercise‐induced regulation of AMPK, p38, and CREB phosphorylation between protocols were not associated with differences in muscle PGC‐1*α* mRNA response.

The current finding that exercise‐induced PGC‐1*α* mRNA response was the same with and without additional arm exercise (C vs. A) despite a marked difference in plasma adrenaline during exercise is in contrast to findings in studies of mice. In mice, injections of adrenaline or beta‐adrenergic agonists induced PGC‐1*α* mRNA in skeletal muscle, whereas beta‐adrenergic antagonists blocked exercise‐induced PGC‐1*α* mRNA response (Miura et al. 2007; Chinsomboon et al. 2009; Tadaishi et al. 2011). It should be noted that the lack of elevated plasma adrenaline at 23 and 39 min of exercise in the protocol with arm exercise may have been a result of the samples being collected 3–4 min following arm exercise.

It cannot be excluded, however, that the period with elevated plasma adrenaline concentrations in the protocol with arm exercise may have been insufficient, and that longer exposure to high‐adrenaline levels may be needed to elevate the muscle PGC‐1*α* mRNA response to exercise. On the other hand, another human study observed no effects of infusion of the nonspecific beta2‐adrenoceptor agonist isoproterenol on mRNA content of PGC‐1*α* or mitochondrial markers (Robinson et al. 2010), suggesting that regulation of the PGC‐1*α* mRNA response differs in humans and rodents (Miura et al. 2007; Chinsomboon et al. 2009; Tadaishi et al. 2011).

Plasma lactate as well as muscle lactate and G‐6‐P levels were higher and muscle CP lower in the sprinting protocols (DS and CS) than the continuous moderate intensity cycling protocols (C and A), which indicates that the metabolic stress was more pronounced in the sprinting protocols. Thus, the observation that PGC‐1*α* mRNA levels were similar after these protocols suggests that metabolic stress is not a determining factor in exercise‐induced PGC‐1*α* mRNA response in human skeletal muscle. It has been observed that reduced muscle glycogen is associated with higher exercise‐induced PGC‐1*α* mRNA levels in human skeletal muscle (Pilegaard et al. 2005; Psilander et al. 2013). However, the observation that differences in muscle glycogen levels between protocols were not associated with differences in PGC‐1*α* mRNA suggests that the level of glycogen is not critical for muscle PGC‐1*α* mRNA response. It should be noted, however, that the muscle glycogen levels differed less than 100 mmol kg dw^−1^ between the protocols in the present study, whereas previous studies reported differences of 200–300 mmol kg dw^−1^ in muscle glycogen (Pilegaard et al. 2005; Psilander et al. 2013). In addition, the muscle PGC‐1*α* mRNA response has been shown to differ between untrained and trained subjects exercising at the same absolute work load without differences in muscle glycogen immediately after exercise (Nordsborg et al. 2010). Taken together, these findings suggest that muscle glycogen level is not a determining factor for exercise‐induced PGC‐1*α* mRNA response in human skeletal muscle.

The present findings that exercise‐induced AMPK, ACC, and CREB phosphorylation differed between the exercise protocols and that PGC‐1*α* mRNA levels were not different, do not support that AMPK and CREB signaling are major mediators of the PGC‐1*α* mRNA response. In contrast, studies in rodents have reported that AICAR treatment (Jorgensen et al. 2005) and CREB‐mediated Ca^2+^ signaling elevated PGC‐1*α* mRNA and protein, respectively, in skeletal muscle, suggesting that AMPK and Ca^2+^ signaling regulates PGC‐1*α* expression. These differences may be due to the experimental set‐up used in the various studies or species differences. Furthermore, of notice is also that both AMPK and p38 have been reported to phosphorylate and thereby activate PGC‐1*α* (Puigserver et al. 2001; Jager et al. 2007), and it is therefore possible that the observed changes in AMPK and p38 phosphorylation elicited differences in PGC‐1*α* activity between the protocols in the present study. However, this speculation remains to be clarified.

The current observation that Cyt c mRNA levels did not change significantly in any of the protocols is consistent with previous studies (Pilegaard et al. 2005; Leick et al. 2010), because mRNA content of various oxidative proteins has been shown to remain unchanged within the initial 6 h of recovery from exercise (Pilegaard et al. 2003), and Cyt c mRNA was first elevated 10 h after exercise (Leick et al. 2010). Nevertheless, numerous studies have reported that PGC‐1*α* regulates the expression of Cyt c in skeletal muscle (Puigserver et al. 2001; Lin et al. 2002; Leick et al. 2008, 2010), which may also have been the case in the present study. The present finding that the overall response of VEGF mRNA resembled the PGC‐1*α* mRNA pattern may suggest that the changes in VEGF mRNA were not elicited by changes in PGC‐1*α* expression. However, it cannot be ruled out that regulation of PGC‐1*α* activity through post translational modifications mediated the observed increase in VEGF mRNA as previously suggested (Jensen et al. 2004).

In conclusion, the present data suggest that differences in plasma adrenaline and muscle metabolic stress during exercise do not reinforce exercise‐induced PGC‐1*α* mRNA response in human skeletal muscle. In addition, differences in exercise‐induced AMPK and p38 signaling were not associated with differences in the PGC‐1*α* mRNA responses, which do not support that AMPK and p38 signaling determines the magnitude of the exercise‐induced PGC‐1*α* mRNA responses in human muscle.

### Study limitations

It is important to mention that the specific time points in the current study may not have caught the actual peak inductions of the different mRNAs due to the transient nature of the responses, and that the timing of the peak may have been different between protocols. For example, the response may be delayed in CS, where sprinting was clustered by the end of the protocol and thus it cannot be ruled out that the enhanced intracellular signaling in CS did affect the PGC‐1*α* mRNA response, but at a time point not investigated. Unfortunately, resolving this issue would require continuous measurements, which clearly is not possible in the current experimental setting. In addition, as described in the statistics section, the variations in mRNA were larger than anticipated.

## Conflict of interest

None declared.
